# An 8-year-old female with a spontaneous tension pneumothorax leading to cardiac arrest complicated by recurrent bronchopleural fistulas requiring endobronchial valves: a case report

**DOI:** 10.1186/s13256-025-05689-y

**Published:** 2025-11-27

**Authors:** Sophia Hartopo, Taylor Marshall, Ivanna Maxson, Justin Greenberg

**Affiliations:** 1 Department of Pediatrics, Mattel Children’s Hospital, University of California, Los Angeles, CA USA; 2 Division of Pediatric Critical Care, Department of Pediatrics, Mattel Children’s Hospital, University of California, Los Angeles, CA USA

**Keywords:** Bronchopleural fistulas, Endobronchial valves, Persistent air leaks, Case report

## Abstract

**Introduction:**

Persistent air leaks, such as bronchopleural fistulas, cause significant health challenges and diagnostic conundrums in both adult and pediatric patients, often delaying recovery and increasing the risk of severe complications. Traditional treatments include prolonged chest tube placement, ventilator manipulation, medical pleurodesis, extracorporeal membrane oxygenation, or surgery, including lobectomy. While used in adult patients, endobronchial valve placement is an emerging, minimally invasive option for treating persistent air leaks in children who have failed other interventions and are not surgical candidates.

**Case presentation:**

This unique case describes an 8-year-old Hispanic female with chronic respiratory failure secondary to tracheobronchomalacia and pulmonary hypoplasia, requiring tracheostomy and lifelong ventilator dependence. She was admitted to the pediatric intensive care unit following cardiac arrest secondary to a tension pneumothorax and subsequently developed recurrent bronchopleural fistulas with multiple pneumothoraces requiring chest tube placement. As part of a nonsurgical management approach, multiple endobronchial valves were placed to address persistent air leaks.

**Conclusion:**

Bronchopleural fistulas are abnormal connections between the bronchial tree and the pleural space, causing persistent air leaks that can complicate lung recovery, especially after lung surgeries, infections, or trauma. Treatment options are varied, and no standardized guidelines exist. This case represents the first reported use of endobronchial valves to treat persistent bronchopleural fistulas in a pediatric patient with chronic lung disease, tracheostomy, and lifelong ventilator dependence, which likely contributed to the recurrence of the bronchopleural fistulas. This case highlights the potential use of endobronchial valves as a minimally invasive alternative to surgery for pediatric patients with persistent air leaks, adding to the current growing literature. It also highlights the challenges faced in this specific pediatric patient population.

## Background

Persistent air leaks (PALs), such as bronchopleural fistulas (BPFs), pose significant diagnostic and therapeutic challenges in both adult and pediatric patients, often prolonging recovery and increasing the risk of severe complications [1]. Conventional management strategies include prolonged chest tube placement, medical pleurodesis, extracorporeal membrane oxygenation (ECMO), and surgical interventions such as lobectomy. While the use of endobronchial valves (EBVs) for PALs is well documented in adults [2, 3], their use in pediatric patients remains limited, especially in children with chronic ventilator dependence, lung hypoplasia, and bronchopleural fistulas. EBVs represent a promising, minimally invasive alternative for children with persistent air leaks who have failed conventional therapies and are not surgical candidates [4].

## Case presentation

**Fig. 1:**
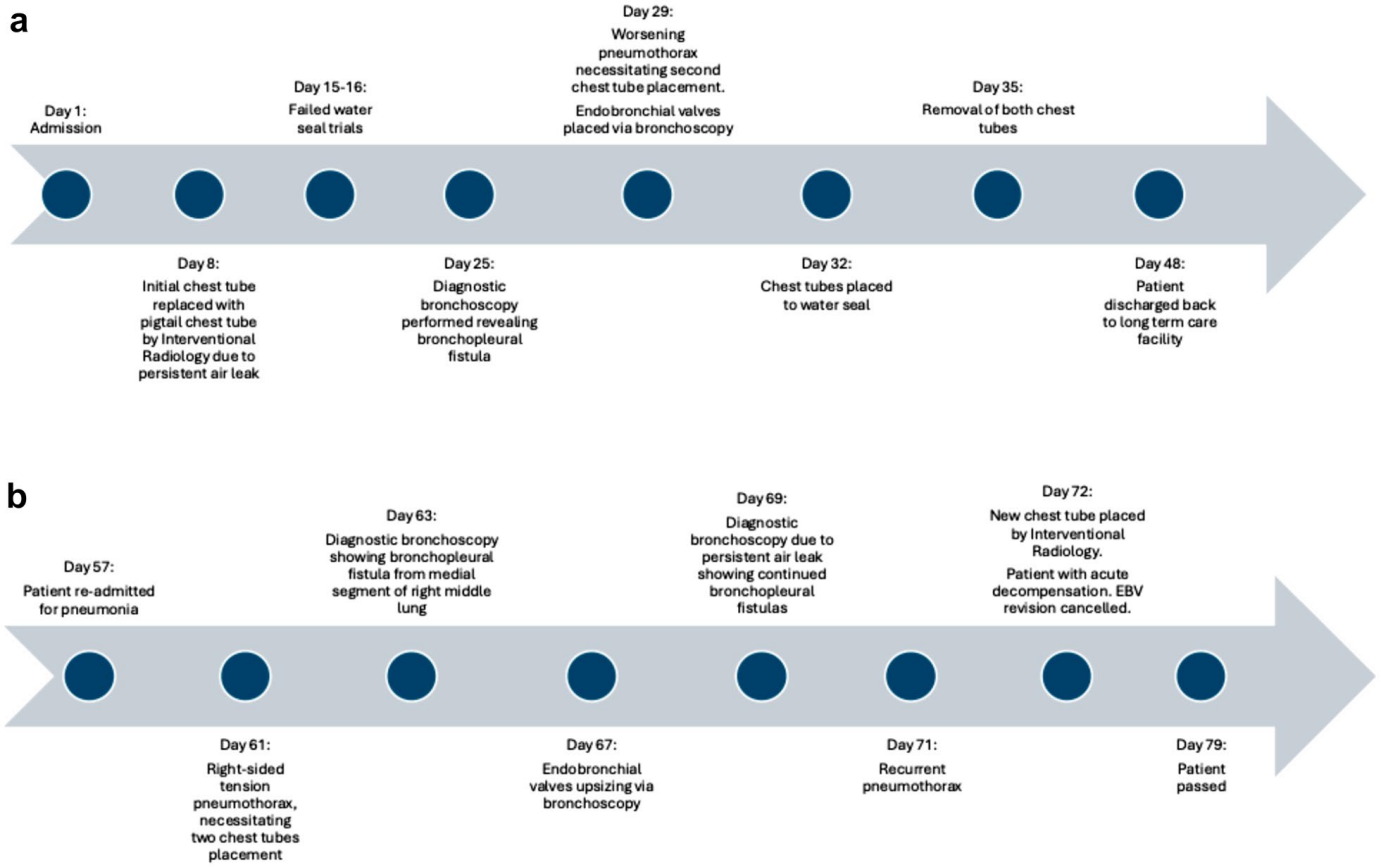
**a**, **b** Timeline depicting patient’s hospital course

We present the case of an 8-year-old Hispanic female with chronic respiratory failure secondary to tracheobronchomalacia and pulmonary hypoplasia, managed with tracheostomy and lifelong ventilator dependence. Her complex medical history included gastrostomy tube dependence, pelvic kidneys with a hypoplastic left kidney, and recurrent ventilator-associated pneumonias. Despite developmental delay, she was ambulatory and able to communicate verbally. She presented to the Emergency Department (ED) with increased work of breathing and oxygen desaturations. On initial examination, she appeared in acute respiratory distress, with nasal flaring, intercostal retractions, and a dusky skin tone. During her ED course, she experienced a cardiac arrest due to a right-sided tension pneumothorax. Return of spontaneous circulation was achieved after 14 min of cardiopulmonary resuscitation and emergent chest tube placement. She was subsequently admitted to the Pediatric Intensive Care Unit (PICU) for ongoing management of her pneumothorax. See Fig. 1a and b for an overview of the patient’s hospital course.

Upon arrival to the PICU, the patient was chemically sedated and paralyzed. Her respiratory examination was notable for diminished expiratory breath sounds. She was initially managed with a peak inspiratory pressure of 35 cm H_2_O and a positive end-expiratory pressure (PEEP) of 5 cm H_2_O, at a respiratory rate of 35 breaths per minute to ensure adequate ventilation while minimizing barotrauma and ventilator-induced lung injury. This was a deviation from her baseline settings of peak pressure 37 cm H_2_O, PEEP 9 cm H_2_O, and rate of 24 breaths per minute.

**Fig. 2:**
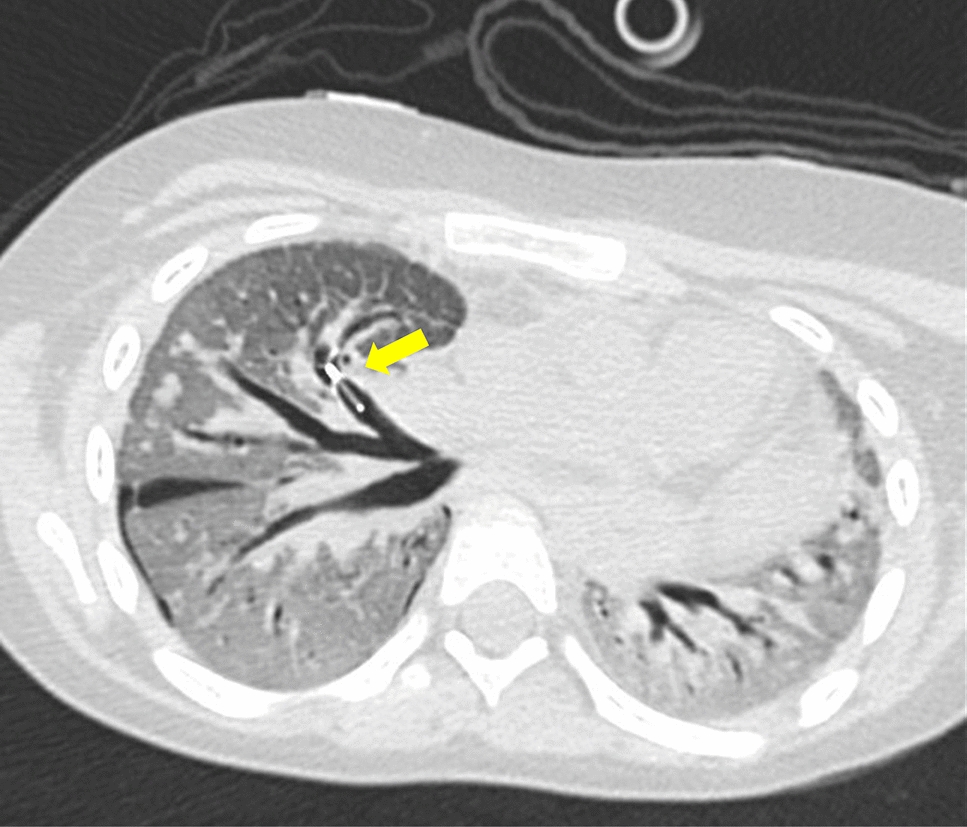
Computed tomography chest image with endobronchial valve in view (yellow arrow)

**Fig. 3:**
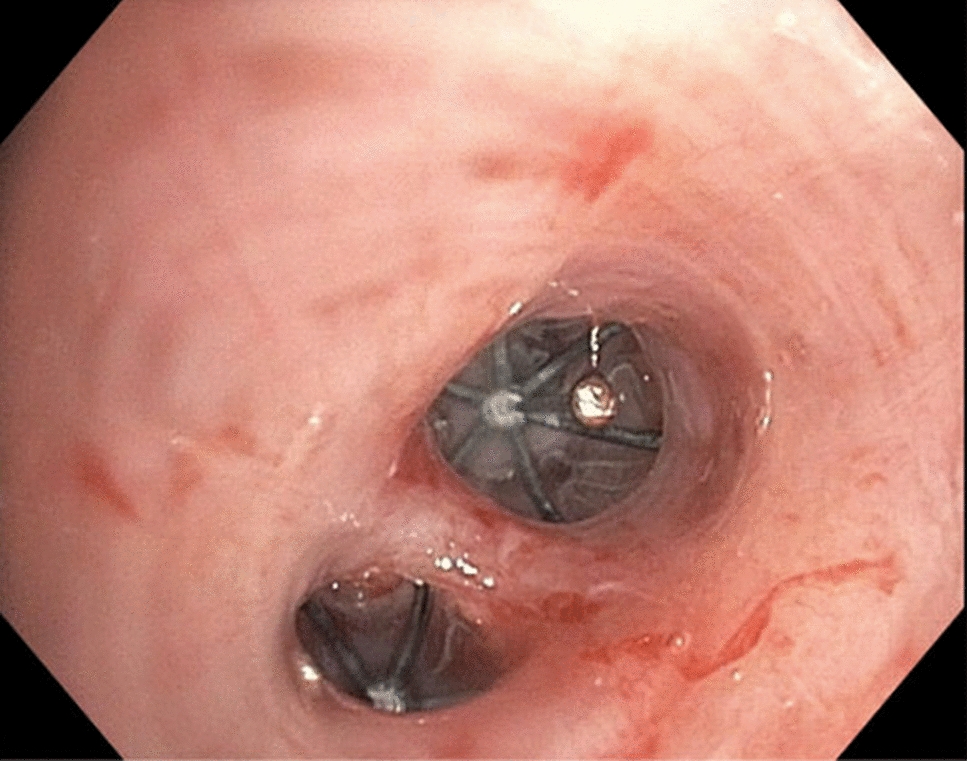
Image of endobronchial valves obtained during bronchoscopy after initial placement. Two valves in view were placed on the medial segment of the right middle lung

Despite initial management, the patient had a persistent air leak, prompting replacement of the chest tube by the Interventional Radiology team; however, the air leak persisted. Bronchoscopic evaluation identified multiple bronchopleural fistulas (BPFs) in the medial segment of the right middle lobe, confirmed by balloon occlusion. Given the complexity of her case, multidisciplinary discussions were held involving pediatric critical care, pediatric surgery, and adult interventional pulmonology. After institutional approval for compassionate use of adult endobronchial valves (EBVs) in a pediatric patient, she underwent bronchoscopy with EBV placement. A 5 mm Spiration^®^ valve was placed in the smaller medial subsegment of the right middle lobe, and a 6 mm valve was placed in the more lateral subsegment (Figs. 2 and 3). No further air leak was noted in the atrium following valve placement. Within three days, both chest tubes were transitioned to water seal and removed 6 days after the procedure without recurrence of pneumothorax or clinical deterioration. The patient was discharged to her long-term care facility on hospital day 48.

At 9 days after her discharge (hospital day 58 from prior admission), the patient returned to the Emergency Department with increased work of breathing and hypoxia. She was diagnosed with pneumonia, and respiratory cultures grew *Pseudomonas aeruginosa* and *Serratia marcescens*. Four days later, she developed another pneumothorax, leading to respiratory decompensation and necessitating chest tube placement. Repeat bronchoscopy revealed that the previously placed endobronchial valves in the medial segment of the right middle lobe had become loose. While the valve in the medial subsegment remained well-positioned, the valve in the lateral subsegment intermittently occluded a subsegmental airway with respiration.

Given these findings, she was considered a candidate for endobronchial valve (EBV) upsizing. Both valves were removed via forceps through the tracheostomy, and an Olympic sizing balloon was used to determine appropriate replacement sizes. The medial subsegment valve was upsized from 5 to 7 mm, and the lateral subsegment valve from 6 to 9 mm. The air leak initially resolved; however, it recurred once the patient resumed spontaneous breathing. Bronchoscopic evaluation confirmed appropriate valve positioning, with no visible air leak adjacent to the valves.

Two days later, a repeat bronchoscopy demonstrated ongoing air leak from the medial subsegment of the right middle lobe. The adult interventional pulmonology team planned a repeat EBV revision using Zephyr^®^ valves. Before this could be performed, the patient suffered another pneumothorax, leading to progressive respiratory failure. She required escalating support, including deep chemical sedation and paralysis, as well as inhaled nitric oxide, to maintain oxygenation and ventilation. Despite maximal medical and ventilatory support, her condition deteriorated with the development of worsening acidosis and acute renal failure. Owing to the severity and irreversibility of her underlying lung disease, she was not a candidate for extracorporeal membrane oxygenation (ECMO), surgical repair, or lung transplantation. She was also deemed too unstable for additional EBV revision or pleurodesis, given high ventilatory pressures and severely impaired lung compliance. Ultimately, her condition continued to decline, and she passed away prior to hospital discharge.

## Discussion

This case underscores the complexities of managing persistent air leaks (PALs) and bronchopleural fistulas (BPFs) in pediatric patients with severe underlying lung pathology. Conventional management strategies—including prolonged chest tube placement, ventilator adjustments, pleurodesis, and surgical interventions—pose significant challenges, especially in patients who are too unstable to tolerate invasive procedures.

In recent years, endobronchial valve (EBV) placement has emerged as a minimally invasive alternative for managing PALs in pediatric patients. A 2012 case study reported EBV use in an 18-year-old patient with cystic fibrosis and PAL as a bridge to lung transplantation [5]. In 2015, the *Journal of Pediatric Surgery* described EBV placement in four pediatric patients with PALs following necrotizing pneumonia, lobectomy, or pneumatocele [6]. In each case, EBV therapy was considered only after failure of all conventional nonsurgical approaches to resolve the air leak.

Additional reports have further demonstrated the potential utility of EBVs in pediatric care. One case described a 16-year-old with pulmonary hemorrhage successfully treated with EBVs and fibrin sealant as a bridge to pneumonectomy [7]. Another involved a 2-year-old with necrotizing pneumonia and refractory acute respiratory distress syndrome (ARDS) requiring ECMO, in whom EBV placement facilitated lung recovery and ECMO decannulation within 9 days [8]. Similarly, an 11-year-old cystic fibrosis patient with pneumothorax on ECMO underwent EBV placement after balloon occlusion failed to resolve the air leak, although the patient ultimately succumbed to complications following lung transplantation [9].

Despite these promising outcomes, EBV use in pediatric patients remains rare, largely limited to cases of ARDS and those requiring ECMO support. Data to guide clinical decision-making remain limited, and therapy must be tailored to the individual, taking into account patient age, underlying pathology, airway anatomy, and ventilatory requirements.

In the present case, EBV placement was pursued owing to the patient’s refractory PALs and ineligibility for surgical or transplant-based interventions. Initial valve placement achieved temporary resolution of the air leak, enabling chest tube removal and discharge. Notably, the patient was able to return to her long-term care facility and celebrate her birthday during the nine days she spent out of the hospital—an outcome that, while not curative, provided her and her caregivers with an invaluable moment of comfort and quality of life.

However, recurrent pneumothoraces and persistent BPFs developed shortly thereafter, highlighting the limitations of this approach in certain high-risk pediatric populations. Our patient’s clinical scenario was unique compared with previously reported pediatric cases of EBV use. Unlike patients with ARDS or those using EBVs as a bridge to lung transplantation, she had chronic ventilator dependence, pulmonary hypoplasia, and a history of recurrent infections and poor lung compliance. We suspect that her high baseline airway pressures, structural lung abnormalities, and the use of adult-sized valves in a pediatric airway contributed to valve loosening without migration and the recurrence of the BPF.

This case illustrates both the potential benefits and the inherent limitations of EBV therapy in pediatric patients with chronic, complex lung disease. It emphasizes the need for further research and device innovation tailored to pediatric airway anatomy, and for clinical frameworks that prioritize not only survival but also meaningful improvements in quality of life.

## Conclusion

This case adds to the growing literature on EBV use in pediatric patients and highlights the need for refining patient selection criteria, assessing long-term outcomes, and developing standardized protocols to optimize EBV implementation in children with complex lung disease. Further research is essential to better define indications, improve procedural success rates, and enhance treatment outcomes in this vulnerable population.

## Data Availability

Not applicable.

## Abbreviations

BPF: Bronchopleural fistula
ECMO: Extracorporeal membrane oxygenation
EBV: Endobronchial valve
COPD: Chronic obstructive pulmonary disease
BLVR: Bronchoscopic lung volume reduction
PAL: Persistent air leaks
